# Exploring Factors Driving the Uneven Distribution of *Aspergillus terreus* in an Austrian Hotspot Region

**DOI:** 10.3390/microorganisms13061218

**Published:** 2025-05-27

**Authors:** Jan Schobert, Paul Illmer, Roya Vahedi-Shahandashti, Cornelia Lass-Flörl

**Affiliations:** 1 ISHAM Working Group of *A. terreus*, ECMM Excellence Center of Mycology, Institute of Hygiene and Medical Microbiology, Medical University of Innsbruck, 6020 Innsbruck, Austria; jan.schobert@i-med.ac.at; 2 Institute of Microbiology, University of Innsbruck, 6020 Innsbruck, Austria; paul.illmer@uibk.ac.at

**Keywords:** *Aspergillus terreus*, environmental distribution, soil, antifungal susceptibility testing, antifungal resistance, aspergillosis, amphotericin B resistance

## Abstract

*Aspergillus terreus* is an opportunistic fungal pathogen and a major cause of aspergillosis. Its clinical significance is heightened by high mortality rates, dissemination, and resistance to amphotericin B, complicating treatment. The present study investigates the distribution of *A. terreus* in Tyrolean (Austria) soils and explores potential environmental factors influencing its uneven prevalence. Soil samples from the eastern and western parts of Tyrol were analyzed using soil extraction plating, the soil immersion tube method, and *A. terreus*-specific qPCR. The results revealed a significantly higher prevalence of *A. terreus* in the eastern region. Soil parameters, including pH, moisture, organic matter, total carbon, and nitrogen, were assessed for potential correlations with fungal distribution. The presence of *A. terreus* was significantly associated with lower pH, decreased total carbon and nitrogen, and lower soil density. Random sampling across Austria indicated a lower environmental frequency of *A. terreus* outside of Tyrol. The susceptibility profiles of amphotericin B, posaconazole, isavuconazole, and voriconazole were determined according to EUCAST guidelines, showing that 98.3% of isolates were wild type for amphotericin B, 100% wild type for voriconazole, 100% resistant to posaconazole, and 87.2% resistant to isavuconazole.

## 1. Introduction

The rise in invasive fungal infections caused by *Aspergillus* species has become a growing concern, particularly among immunocompromised patients, due to the associated high morbidity and mortality [1,2]. Following *A. fumigatus*, the most frequently isolated species, *A. terreus* is one of the leading causes of invasive aspergillosis (IA) [3,4,5]. The clinical significance of *A. terreus* lies primarily in its elevated in vitro minimum inhibitory concentration (MIC) to amphotericin B, along with its strong association with high rates of dissemination and mortality (ranging from 51% to 70%) in cases of IA [4,6].

*A. terreus* is recognized as a prominent cause of IA in several clinical settings, including the M. D. Anderson Cancer Center in Houston, TX, USA, and the University Hospital of Innsbruck, Austria [7,8]. Considering the aforementioned clinical significance of *A. terreus*, together with its high incidence at the University Hospital of Innsbruck, Austria, and its uneven environmental distribution across Tyrol, the federal state in which the hospital is located, our research group was prompted to investigate the underlying factors contributing to this increased prevalence [9,10,11]. Dietl et al. (2021) reported significant variation in the environmental distribution of *A. terreus* across Tyrol, Austria, with isolates clustering into three distinct genotypes [10]. Their study also found a markedly higher occurrence in the eastern part of Tyrol compared to the west, identifying agricultural cornfields as a key environmental reservoir [10]. Moreover, the environmental prevalence of *A. terreus* demonstrated a strong correlation with the increased incidence of infections in specific geographical regions [10]. This correlation was further supported by a serosurveillance study measuring *A. terreus* IgG antibody levels, which revealed a significant difference between the western and eastern part of Tyrol [12].

However, the precise factors underlying this distribution pattern remain to be elucidated, which prompted the present study to investigate the potential environmental influences in greater depth. As Tyrol has distinct geographical and climatic characteristics, its eastern and western regions could each potentially harbor different environmental conditions that shape fungal ecology, thereby necessitating a detailed investigation into potential micro- and macro-niche differentiation. Besides its specific environmental distribution, treating invasive fungal infections (IFIs) caused by *A. terreus* is challenging [13], due to its reduced susceptibility to amphotericin B [14,15] and the rising rates of resistance to azoles, the first-line therapy [16,17,18]. This underscores the need for continuous monitoring of antifungal susceptibility profiles of this species, particularly in regions with high prevalence, to facilitate the establishment of local epidemiological cut-off values (ECOFFs).

As demonstrated by Dietl et al. (2021), soil served as the primary environmental reservoir for isolating *A. terreus* [10]; building on this, the present study aims to provide an updated assessment of *A. terreus* distribution and to investigate how soil physicochemical properties may contribute to the observed differences between high- and low-distribution areas in the eastern and western regions. The second objective is to assess how different soil sampling and detection methods may influence the evaluation of *A. terreus* distribution. This includes a comparative analysis of direct DNA extraction from soil followed by *A. terreus*-specific qPCR, the soil immersion tube method, and the conventional soil extraction plating technique. The third objective is to broaden the geographical scope of the investigation by assessing the environmental presence of *A. terreus* in other Austrian regions through randomized soil sampling. The final objective is to update the antifungal susceptibility profiles of environmental *A. terreus* isolates from Tyrol, with a focus on identifying emerging resistance patterns relevant to clinical treatment strategies and enhancing preparedness efforts.

## 2. Materials and Methods

### 2.1. Sampling Scheme and Sampling Area

A total of 80 agricultural soil sampling sites, 40 each in the eastern and western regions, previously identified as key environmental reservoirs [10], were selected for a one-year analysis of *A. terreus* occurrence and distribution in Tyrol, Austria. Sampling was conducted at each location once per season between March 2023 and February 2024, resulting in a total of 320 individual soil samples. In addition to the soil sampling conducted in Tyrol, a random analysis of soil samples from all Austrian federal states was performed to assess the presence of *A. terreus* beyond the Tyrolean region.

### 2.2. Soil Sampling

A pooling strategy was employed at each sampling location to obtain soil samples representative of a broader site condition. Five randomly selected points within each site were combined to form a composite sample. From each point, the uppermost soil layer was removed, and 5–10 g of soil were collected using a disinfected shovel and added to the pooled sample. Composite samples were placed in sterile freezer bags and stored at 4 °C until further processing. Prior to analysis, all samples were sieved through a 2-mm mesh to remove coarse particles. The presence of *A. terreus* was assessed using both the soil extraction plating method and *A. terreus*-specific qPCR. Additionally, soil physicochemical properties—including pH, moisture content, organic matter, total carbon, and nitrogen—were analyzed.

### 2.3. Soil Properties

To measure soil pH, 5 g of each sieved soil were vortexed in 12.5 mL of 0.01 M CaCl_2_ and allowed to settle for at least two hours. After re-mixing, the pH was measured using a pH 526 meter (WTW, Weilheim, Germany). The soil moisture was determined by measuring mass loss after drying at 105 °C until a constant weight was reached, with the difference between the moist and dry weights used to calculate moisture percentage. The dried soils were subsequently used for organic matter analysis through loss-on-ignition, involving ashing at 430 °C for five hours. The weight loss before and after ashing was measured to estimate organic matter content. The total soil carbon and nitrogen were quantified using a CN-analyzer (CN828, Leco, St. Joseph, MI, USA) according to standard protocols.

### 2.4. Soil Extraction Plating Method

To determine the presence of *A. terreus* in the soil samples, a soil extraction plating method was applied, as described previously based on Dietl et al. (2021) [10]. Each sample was tested in duplicate. For each replicate, 2 g of sieved soil was mixed with 5 mL of spore buffer (0.9% NaCl, 0.1% Tween 20 in 1000 mL deionized water) in a 15 mL Falcon tube, followed by vortexing at maximum speed for 1 min. After allowing the mixture to settle for 3 min, 100 µL of the supernatant was plated onto Sabouraud Agar medium (bioMérieux, Marcy l’Etoile, France) supplemented with 50 mg L^−1^ chloramphenicol (Sigma-Aldrich, Vienna, Austria) to inhibit bacterial growth, and 0.5 mg L^−1^ of amphotericin B (0000122836, Sigma-Aldrich, Vienna, Austria) to suppress the growth of non-target fungal species. For each soil extraction, a total of four agar plates were prepared and incubated for 48 to 72 h. Three plates contained Sabouraud dextrose agar supplemented with chloramphenicol, while the fourth additionally included amphotericin B. To exclude non-thermotolerant organisms, one chloramphenicol-supplemented plate was incubated at 50 °C, while the remaining plates were incubated at 37 °C. Following incubation, plates were examined for colonies with morphological characteristics suggestive of *A. terreus*. Suspected colonies were subcultured onto oatmeal agar (15 g edible crushed oatmeal, 7.5 g agar (22C2856629, VWR Chemicals, Solon, OH, USA), 500 mL deionized water) for macroscopic and microscopic identification.

### 2.5. Soil Immersion Tube Method

**Figure 1:**
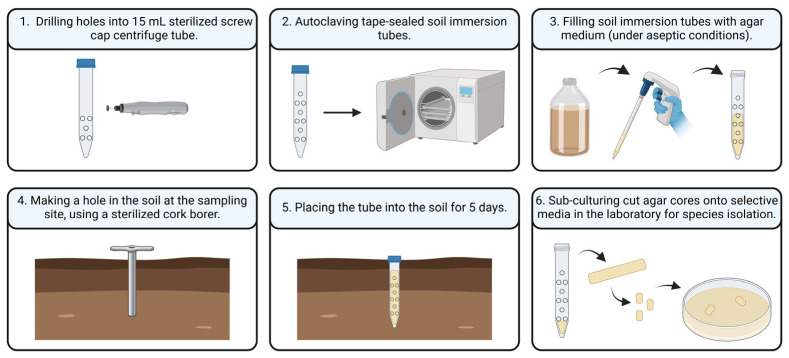
Schematic illustration of the soil immersion tube method.

The soil immersion tube method (Figure 1), adapted from Chesters [19], was used to assess the presence of actively growing hyphae in the soil at the sampling sites. This method was carried out during the summer season, as favorable weather conditions during this period facilitated the implementation of the procedure. For this study, standard 15 mL Falcon tubes were modified by drilling ten holes into each tube and were sterilized by autoclaving. Before adding the agar medium (Sabouraud supplemented with chloramphenicol and amphotericin B), the modified tubes were sealed with parafilm to prevent leakage. At each sampling site, a hole matching the diameter of the soil immersion tubes was drilled into the ground. The tubes, after removal of the parafilm, were then inserted into the soil and incubated in situ for approximately five days. Following the incubation period, the agar core from each soil immersion tube was aseptically cut into sections and subcultured on Sabouraud agar plates to determine the presence of *A. terreus*.

### 2.6. DNA Extraction and qPCR

A total of 120 soil samples (30 sampling sites, 15 per region, for all four seasons) were tested for the presence of fungal DNA using *A. terreus*-specific qPCR. DNA extraction was performed using the sieved soil, following the manufacturer’s protocol with the NucleoSpin Soil Kit (Machery-Nagel, Düren, Germany). The qPCR analysis targeted the *Aspergillus terreus* glucose dehydrogenase gene and was performed using the genesig Advanced Kit (Primerdesign, Eastleigh, UK) with the included *A. terreus* specific primers according to the manufacturer’s instructions.

### 2.7. Publicly Available Data for Tyrol

In order to analyze the distribution patterns of *A. terreus*, publicly available data for Tyrol were utilized alongside the data collected in this study. Soil data from the Austrian online soil map eBod (https://bodenkarte.at, accessed on 20 June 2024), including information about soil density, weather data from GeoSphere Austria (https://data.hub.geosphere.at/dataset/inca-v1-1h-1km, accessed on 18 December 2024) and population data for Tyrol from Statistics Austria (https://www.statistik.at/atlas/, accessed on 18 December 2024) were examined.

### 2.8. Antifungal Susceptibility Testing

The *A. terreus* isolates obtained were tested for antifungal susceptibility according to the EUCAST guidelines (version 9.4, https://www.eucast.org/astoffungi/methodsinantifungalsusceptibilitytesting/ast_of_moulds, accessed on 11 July 2024) [20]. Antifungal susceptibility testing was performed for the following agents: amphotericin B (0000122836, Sigma-Aldrich, Vienna, Austria), voriconazole (20322, MedChemExpress Europa, Sollentuna, Sweden), posaconazole (SML 2287, Sigma-Aldrich, Vienna, Austria) and isavuconazole (SML 2357, Sigma-Aldrich, Vienna, Austria).

### 2.9. Data Analysis and Statistics

The geographical maps were generated using the free and open-source software QGIS (QGIS 3.34.12-Prizren, https://qgis.org, accessed on 18 December 2024), with the map of Tyrol derived from the Land Tirol dataset [21]. The graphic creation and data analysis were performed with Origin (OriginPro 2024, OriginLab Corporation, Northampton, MA, USA).

## 3. Results

### 3.1. Distribution of A. terreus Across Tyrol

**Figure 2:**
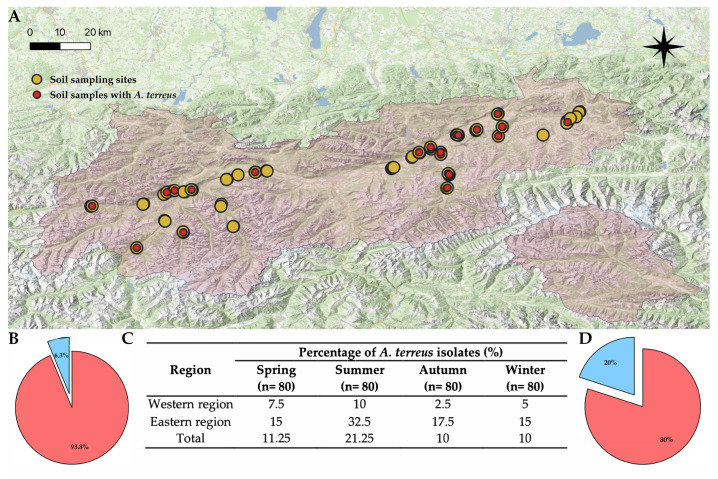
Map of Tyrol showing the distribution of soil sampling sites (yellow dots, soil extraction plating method), including those in which *A. terreus* was found (red dots) (**A**). Pie chart of *A. terreus* frequency in the western part of the sampling region (in blue) (**B**). Table of seasonal *A. terreus* occurrence in the sampling region (**C**). Pie chart of *A. terreus* frequency in the eastern part of the sampling region (in blue) (**D**).

**Figure 3:**
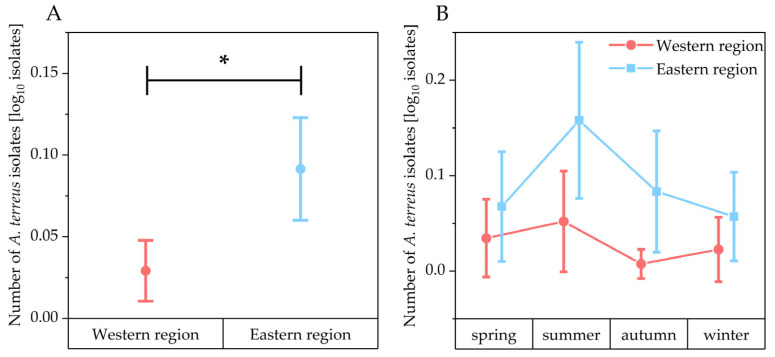
Number of *A. terreus* isolates (log_10_ transformed) in the soil samples. Comparison between the western and eastern region (* indicates *p* < 0.05, Mann–Whitney test: z = −3.60 *p* < 0.05) (**A**). Seasonally sorted comparison between the western and eastern region (**B**). Data points represent means and error bars represent 95% confidence intervals. Samples from the western region are indicated in red and samples from the eastern region are indicated in blue.

Out of 320 soil samples collected in one year, *A. terreus* was detected in 42 samples (13.1%) (Figure 2). In all four seasons, the proportion of *A. terreus*-positive samples was consistently higher in the eastern part of the study region compared to the west (Figure 3). This difference was statistically significant for the total sampling period (Mann–Whitney test: z = −3.60 *p* < 0.05). The highest number of *A. terreus*-positive samples in both regions was recorded during the summer.

### 3.2. Comparison of Soil Immersion Tube Method and A. terreus Specific qPCR

Of the eighty soil immersion tubes tested during the summer sampling period, *A. terreus* was isolated from seven tubes, five from the eastern region and two from the western region. All *A. terreus* isolates recovered using the soil immersion tube method originated from sampling sites where *A. terreus* was also detected using the conventional soil extraction plating method, with the exception of a single isolate from the western region that was exclusively detected by the immersion tube method. Out of the 120 qPCR reactions conducted across 30 sampling sites, only 5 reactions showed amplification. Comparative analysis of three detection methods revealed that *A. terreus* was most frequently identified using the conventional soil extraction plating method. The soil immersion tube method is significantly comparable to regular sampling; in summer (Mann–Whitney test: z = −6.86 *p* < 0.05), *A. terreus* was found in 32.5% of the samples from the eastern sampling region and in 12.5% of the soil immersion tubes. In the western region, *A. terreus* was identified in 10% of the samples obtained through regular sampling during the summer months, and in 5% of the soil immersion tubes. Nevertheless, the qPCR results of this study demonstrated no correlation with the results obtained from the other two methods.

### 3.3. Physicochemical Soil Parameters

**Figure 4:**
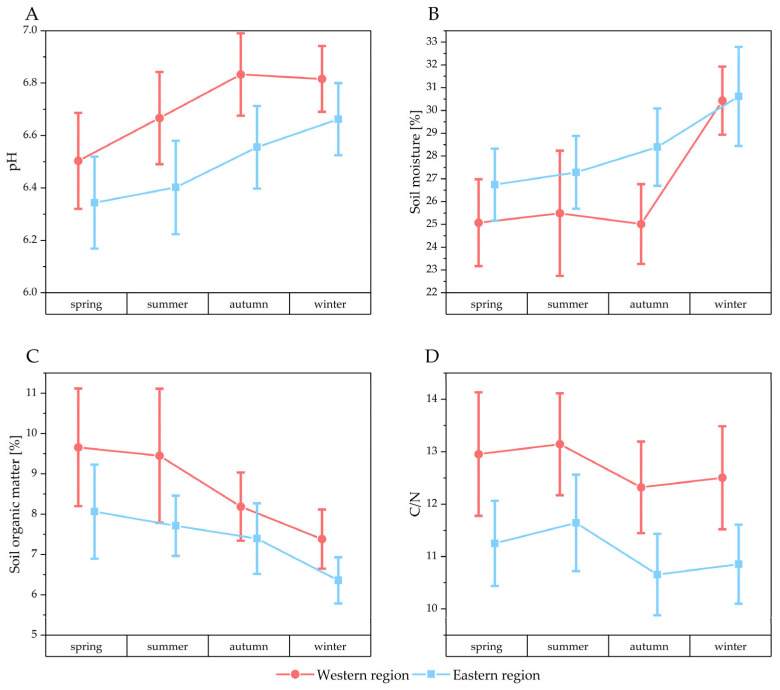
Seasonally sorted physicochemical properties of the soil samples including pH (**A**), soil moisture (**B**), soil organic matter (**C**) and C/N ratio (**D**). Data points represent means and error bars represent 95% confidence intervals. Samples from the western region are indicated in red and samples from the eastern region are indicated in blue.

Figure 4 presents the soil parameters measured in this study, which have been organized by season and region. In addition, these data are available in the Supplementary Material, where they are sorted by soils with and without *A. terreus* (Figure S1). Supplementary plots for total carbon and nitrogen levels are also provided (Figure S2).

The pH value of the soil samples from the eastern region (median: 6.64) was slightly but significantly lower than that of the soil samples from the western region (median: 6.86) (Mann–Whitney test: z = 4.45 *p* < 0.05). Additionally, a significant disparity in pH was observed between soil samples with (median: 6.66) and without *A. terreus* (median: 6.8) (Mann–Whitney test: z = 2.07 *p* < 0.05). Furthermore, *A. terreus* was found to be more prevalent in soils with slightly higher acidity levels (Figure S1). Soil moisture exhibited a slight but statistically significant difference between the eastern and western sampling regions, with the eastern region showing a higher median value (28.1) compared to the western region (26.8) (Mann–Whitney test: z = –2.52, *p* < 0.05). However, no significant difference in soil moisture was observed between samples with *A. terreus* (median: 26.5) and those without (median: 27.8) (Mann–Whitney test: z = 0.75, *p* = 0.45).

Soils from the western region (median: 7.6) had significantly higher organic matter content than those from the eastern region (median: 7.2) (Mann–Whitney test: z = 2.66, *p* < 0.05). Additionally, soils without *A. terreus* (median: 7.6) had significantly higher organic matter content than soils harboring *A. terreus* (median: 6.8) (Mann–Whitney test: z = 1.98, *p* < 0.05).

Total carbon was significantly higher in the western sampling area (median: 4.78) compared to the eastern part (median: 3.76) (Mann–Whitney test: z = 5.83 *p* < 0.05). Similarly, higher median values were observed in soils without *A. terreus* (median: 4.31) compared to soils with *A. terreus* (median: 3.76) (Mann–Whitney test: z = 2.08 *p* < 0.05).

Total nitrogen levels were significantly higher in the western (median: 0.375) than in the eastern region (median: 0.336) (Mann–Whitney test: z = 2.9 *p* < 0.05). However, there was no significant difference in total nitrogen levels between soils containing *A. terreus* (median: 0.306) and those without it (median: 0.366) (Mann–Whitney test: z = 2 *p* = 0.051).

The western part (median: 11.5) exhibited a significantly higher carbon to nitrogen (C/N) ratio than the eastern share of the sampling region (median: 10.3) (Mann–Whitney test: z = 5.5 *p* < 0.05). No significant difference was observed in the C/N ratio between soils with (median: 10.4) and without *A. terreus* (median: 10.9) (Mann–Whitney test: z = 0.94 *p* = 0.34).

The seasonally indexed soil parameter data demonstrate that, while there is seasonal variance for all measured parameters, the overall pattern and the difference between the two parts of the sampling region remain relatively consistent throughout the sampling period. For instance, while soil moisture fluctuates over the year and is significantly higher in winter compared to other seasons in both regions (KWANOVA: X^2^ (df = 3, n = 320) = 31.41, *p* < 0.05), it consistently remains higher in the eastern part than in the western part across all seasons (Mann–Whitney test: z = −2.52 *p* < 0.05).

### 3.4. Analysis of Publicly Available Data and Factors Influencing the Distribution Pattern of A. terreus

Of the 80 sampling sites, 50 were in maize fields, 16 in grasslands, 7 in potato fields, 2 in onion fields, and 1 in a rye field. Four sites were uncultivated. No significant difference in *A. terreus* occurrence was found between maize and grassland fields.

Beyond soil parameters (Section 3.3), additional factors were analyzed for their impact on *A. terreus* distribution using the Austrian soil database eBod [22]. The fungus was significantly more frequent in less dense soils (Χ^2^(df = 4, n = 316) = 15.12, *p* < 0.05). No significant correlation between the presence of *A. terreus* and air temperature or humidity during the sampling activity was found using weather data from GeoSphere Austria [23].

Population density and tourism were analyzed using STATAtlas [24]. While tourism levels were similar across regions, population density was significantly higher in the eastern part of the sampling region (Mann–Whitney: z = −11.38, *p* < 0.05). *A. terreus* was more frequent in areas with higher population density, including the western region (Supplementary Material, Figure S3).

Spearman correlation analysis indicated that population density had the strongest but weak correlation with *A. terreus* occurrence (r(318) = 0.244, *p* < 0.05). The fungus was more abundant in less dense, acidic soils with lower total carbon and nitrogen levels, as indicated by weak negative correlations with soil heaviness (r(318) = −0.184), pH (r(318) = −0.131), total carbon (r(318) = −0.123), and total nitrogen (r(318) = −0.112) (all *p* < 0.05). However, soil organic matter (r(318) = −0.108, *p* = 0.054) and air temperature (r(318) = 0.106, *p* = 0.057) demonstrated no significant correlation at *p* < 0.05.

### 3.5. Distribution of A. terreus Across Austria

**Figure 5:**
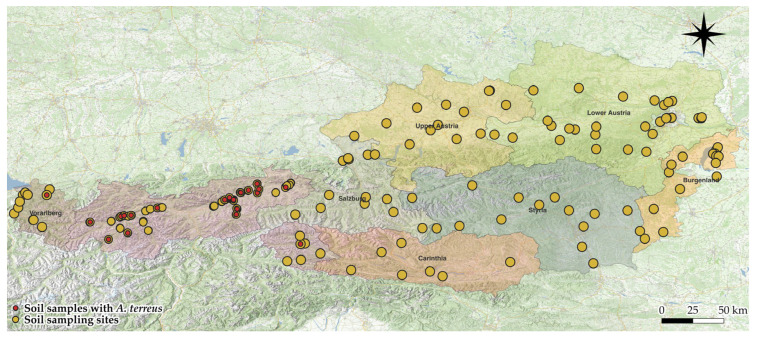
Distribution of soil samples with and without *A. terreus* across Austria.

A total of 111 random soil samples were collected in Austria, excluding Tyrol (including 6 from East Tyrol) (Figure 5). *A. terreus* was detected in only one sample from East Tyrol and one from Vorarlberg. No *A. terreus* isolates were isolated from the other federal states—Salzburg, Carinthia, Upper Austria, Lower Austria, Styria, Vienna, and Burgenland. Overall, the detection rate of *A. terreus* outside of Tyrol was 0.95%.

### 3.6. Antifungal Susceptibility Testing

**Figure 6:**
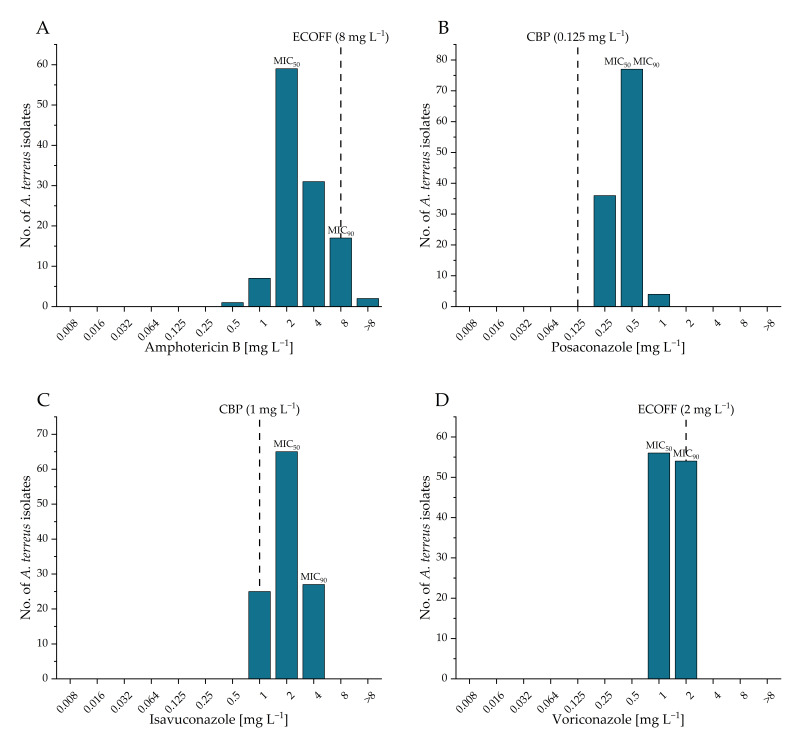
Minimal inhibitory concentrations distribution of western and eastern Tyrolean *A. terreus* isolates against amphotericin B (**A**), posaconazole (**B**), isavuconazole (**C**) and voriconazole (**D**). The MIC for 50% and 90% of the tested population is marked. For amphotericin B and voriconazole the Epidemiological Cut-Off Value (ECOFF) is shown, for posaconazole and isavuconazole the clinical breakpoint (CBP) (https://www.eucast.org/astoffungi/clinicalbreakpointsforantifungals, accessed on 8 February 2025) [25].

The MICs distribution of four tested antifungal agents—amphotericin B, posaconazole, isavuconazole, and voriconazole—are shown in Figure 6, alongside their respective ECOFFs and clinical breakpoints (CBPs) as defined by EUCAST (https://www.eucast.org/astoffungi/clinicalbreakpointsforantifungals, accessed on 8 February 2025) [25]. MIC of amphotericin B ranged from 0.5 to >8 mg L^−1^, with 98.3% of the tested isolates categorized as wild type according to the EUCAST ECOFF. All tested isolates were resistant to posaconazole according to EUCAST CBPs, with MICs ranging from 0.25 to 1 mg L^−1^. In total, 87.2% of isolates demonstrated Isavuconazole resistance, with MICs ranging from 1 to 4 mg L^−1^, based on the EUCAST CBPs. In contrast, all *A. terreus* isolates were wild type to voriconazole, with MICs ranging from 1 to 2 mg L^−1^ [24]. No significant differences were observed in the antifungal susceptibility profiles of *A. terreus* isolates between the western and the eastern part of the sampling region (Mann–Whitney tests: amphotericin B: z = 0.23; posaconazole: z = 0.53; isavuconazole: z = 0.30; voriconazole: z = 0.54; all *p* > 0.05). The actual MIC values for each isolate are provided in the Supplementary Material (Table S1).

## 4. Discussion

A previous study conducted by Dietl et al. (2021), involving a year-long soil sampling in Tyrol, Austria, demonstrated that soil serves as the primary environmental niche and stable reservoir for *A. terreus*, with a significantly higher occurrence in the eastern part of the region compared to the west [10]. These findings aligned with the clinical data and the significantly different levels of *A. terreus*-specific IgG antibodies observed between the eastern and western part of the region [12]. This prompted the current investigation, which aimed to further explore this uneven distribution and identify the specific influencing factors contributing to the prevalence of differences between these two regions. The present study reaffirms previous findings of a higher prevalence of *A. terreus* in the eastern region. Nevertheless, in contrast to the findings of Dietl et al. (2021), which found higher frequencies in the cold season, the majority of *A. terreus* isolates in our study were isolated during the summer [10]. Different seasonal occurrence patterns of *A. terreus* have been reported also in other regions [26,27]. For instance, a study by Guinea et al. (2006) in Madrid, Spain, found the highest frequency of *A. terreus* in autumn [26]. Similarly, a study by Ebner et al. (1989) on airborne spores in the Inntal observed peaks of *Aspergillus* spore counts in both summer and winter [27]. The exact reasons for the shift from the winter prevalence of *A. terreus* isolates reported by Dietl et al. (2021) to the summer predominance observed in the present study remain unclear [10]. Potential causes of the observed differences include variations in climatic conditions and differences in resource availability regimes between the two sampling periods [28,29]. For example, different fertilization regimes between years can influence the diversity and abundance of fungi in soil [30]. Additionally, seasonality was a limitation of this study, as sampling began in the cold season, which precluded a comprehensive evaluation of the impact of crop selection on fungal distribution.

A key finding from Dietl et al. (2021) was that soil serves as the main reservoir for *A. terreus* [10]. In this study, comparative screening methods were employed to determine the most effective approach, using the same soil extraction plating method as Dietl et al. (2021) [10], along with soil immersion tubes and direct DNA extraction from soil. The soil extraction plating method and soil immersion tubes were comparably effective in detecting *A. terreus* in soil samples, although the immersion tube method demonstrated lower sensitivity. In contrast, direct DNA detection from soil yielded the fewest positive results. This discrepancy may be due to the fact that, although PCR is a highly sensitive method, the amount of fungal material must be sufficient to detect it [31,32], and perhaps not enough sample material was present in the soil samples analyzed, e.g., due to uneven distribution of the fungal material in the soil [31]. The utilization of the soil immersion tube method has also demonstrated the active growth of *A. terreus* hyphae within the soil environment [19].

The distribution of fungi in general and *A. terreus* could be influenced by several environmental factors, these include soil pH, total carbon and nitrogen content, and soil density [33,34,35]. *A. terreus* is known to thrive across a wide pH range and has been commonly found in soils with a pH of approximately 5–6 [36,37,38], indicating a preference for slightly acidic conditions [37,38]. Our study similarly detected *A. terreus* more frequently in neutral to slightly acidic soils, with a negative correlation between its presence and soil pH. This aligns with findings that certain filamentous fungi favor acidic environments, as pH influences fungal enzymatic activity, sporulation, and competitive interactions within microbial communities [39].

The occurrence of *A. terreus* in Tyrolean soils showed a negative correlation with total carbon and nitrogen levels, indicating a higher prevalence in soils with comparatively lower nutrient levels. *Aspergillus* species, in general, have been reported to have a positive correlation with organic matter content [40,41]. A study conducted in the Negev Desert found that *A. fumigatus* and other thermotolerant fungi were more abundant in areas with higher organic matter levels [41]. In this study, the median organic matter level in all soils tested was found to be relatively high [42]. Consequently, these soils seem conducive to the proliferation of saprophytic fungi [43]. Therefore, the increased frequency of *A. terreus* in the eastern region cannot be explained by the nutrient content of the soils themselves. This is further supported by *A. terreus*’s ability to utilize a wide range of carbon and nitrogen sources [44]. Additionally, the lower soil density measured in our study is known to facilitate spore dispersal and colonization of *A. terreus* due to improved aeration and easier movement of the fungus within the soil [33,45]. While we analyzed key soil chemical properties, additional factors such as microbial community composition were not examined. Given that soil microbiota interactions can influence fungal prevalence [46,47], further studies integrating metagenomic and metabolomic approaches may provide deeper insights into the ecological drivers of *A. terreus* distribution. Soil moisture and weather conditions, which are also known to influence fungal growth and distribution [48,49,50], were therefore analyzed in this study; however, no significant correlation was found between the occurrence of *A. terreus* in Tyrol and these factors.

The randomly selected soil samples from across Austria showed that *A. terreus* is significantly less prevalent outside of Tyrol, suggesting that regional environmental conditions play a role in shaping its distribution. Studies of soil fungal ecology have indicated that factors such as microclimate, soil moisture, organic matter, and local vegetation contribute to the regulation of fungal diversity and abundance [33,35,51]. The absence of *A. terreus* in most other regions suggests that its prevalence in Tyrol may be related to unique geographic, ecological, or human-related factors, such as valley landscapes or specific agricultural practices warranting further investigation. Despite the considerable sample size, limited sampling outside Tyrol may have introduced bias, leading to an imbalance that could influence the broader interpretation of *A. terreus* distribution in Austria. This uneven distribution highlights the need for more extensive and geographically representative sampling in future studies to accurately assess the species’ distribution and environmental drivers.

Amphotericin B and voriconazole susceptibility profiles exhibited consistent results with previous studies, with all isolates categorized as wild type [10,52,53]. *A. terreus* is commonly regarded in the literature as intrinsically resistant to amphotericin B [15,54,55]. However, previous studies have demonstrated that not all *A. terreus* isolates exhibit amphotericin B resistance [6,56,57]; some are susceptible or tolerant to amphotericin B. The broad range of amphotericin B MICs among *A. terreus* isolates, ranging from 0.5 to 8 mg/L, complicates the establishment of definitive ECOFFs and CBPs for this species. Additionally, the narrow concentration ranges used in susceptibility testing may not fully capture this variability, further hindering accurate susceptibility assessments. Consequently, the terms “innate” or “intrinsic” amphotericin B resistance should be applied with caution when referring to this species.

Alarmingly, all of the tested isolates showed resistance to posaconazole (100%), and 87.2% were resistant to isavuconazole. The observed trend of increased MICs for posaconazole could indicate a potential shift in antifungal susceptibility patterns among isolated *A. terreus* [10]. The MIC_90_ of posaconazole for *A. terreus* is generally reported at 0.5 mg L^−1^ [58,59], which is consistent with the present study. However, regarding categorization, previous studies have demonstrated posaconazole resistance in *A. terreus* ranging from 5% to 25% [10,16], while in the present study 100% of the isolates were grouped as resistant. This shift in trend could be attributed to the recent change in the EUCAST guidelines, which revised the CBP for posaconazole from 0.5 to 0.125 mg L^−1^ [25]. However, further investigation is needed to determine whether this trend is linked to only phenotypic resistance or real genetic mutations, causing higher posaconazole susceptibility levels. A comparison of the isavuconazole MICs from the present study with those reported in other studies indicates elevated levels; for instance, Espinel-Ingroff et al. (2013) reported that the MIC_90_ for *A. terreus* is typically 1 mg L^−1^ [60,61]. Nevertheless, it is noteworthy that Jorgensen et al. (2019) has also reported elevated isavuconazole MICs for *A. terreus* [62].

Environmental exposure to agricultural azoles, as fungicides, could contribute to the development of acquired resistance by exerting selective pressure [63]. Since the early 2000s, there has been a consistent and gradual increase in the utilization of azoles in agriculture [64], promoting the survival and proliferation of resistant *Aspergillus* mutants, particularly those carrying mutations such as TR_34_/L98H or TR_46_/Y121F/T289A in the *cyp*51A gene, which are known to confer cross-resistance to medical azoles [65,66].

Studies have shown that mutations in the *cyp*51A gene, are frequently associated with resistance and correlate with widespread use of azole fungicides in agriculture [65,67], which can also lead to cross-resistance to other azoles, complicating the treatment of aspergillosis [63]. Although *A. terreus* has not been as extensively studied in this context, it is plausible that similar selective pressures may contribute to the development of azole resistance in environmental isolates, warranting further investigation.

## 5. Conclusions

This study provides a comprehensive update on the distribution of *A. terreus* in a distinct hotspot region of Austrian soils. The findings indicate a higher prevalence of the species in the eastern part of the region, with a seasonal shift from higher occurrence in winter to summer. Soil remains a primary reservoir for *A. terreus*, and we have found that soil extraction plating and immersion tube methods were more reliable than direct DNA analysis for detecting the fungus. The present study found that environmental factors, such as pH and soil density, exert influence on the prevalence of *A. terreus* in soil, while no significant correlation was found with soil moisture or weather conditions. The species was found to be less prevalent in other Austrian regions, thereby highlighting Tyrol’s unique role as a hotspot for *A. terreus*. Antifungal susceptibility testing has demonstrated an increase in resistance of *A. terreus* to posaconazole and isavuconazole. This phenomenon is likely associated with environmental exposure to azoles, underscoring the necessity for further investigation and enhanced preparedness.

## Data Availability

The authors confirm that all data/protocols are detailed within the article and Supplementary Files.

## Supplementary Materials

The following supporting information can be downloaded at: https://www.mdpi.com/article/10.3390/microorganisms13061218/s1, Figure S1: Physicochemical properties of the soil samples; Figure S2: Total carbon and nitrogen of the soil samples; Figure S3: Population density of the sampling sites; Table S1: Minimal inhibitory concentrations of environmental *A. terreus* isolates.
