# Supplementary material for: Exploring Factors Driving the Uneven Distribution of Aspergillus terreus in an Austrian Hotspot Region
Source: Microorganisms. 2025 May 27;13(6):1218. doi: 10.3390/microorganisms13061218 (PMC12195318; doi:10.3390/microorganisms13061218)
Supplement: Supplementary file 1 [file microorganisms-13-01218-s001.zip › Figure S1.pdf]

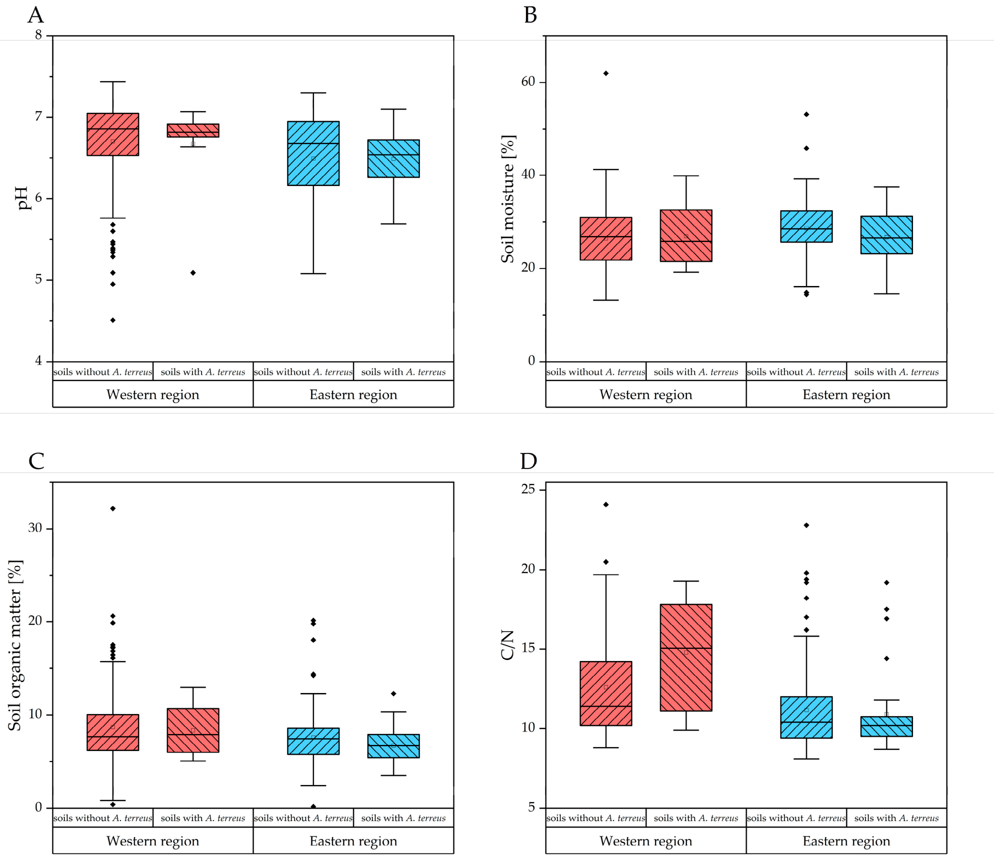

Figure S1. Physicochemical properties of the soil samples including pH (A), soil moisture (B), soil organic matter (C) and C/N ratio (D). Boxes represent 25-75% of values, black lines medians, whiskers 1.5 interquartile ranges, hollow squares means and diamonds outliers. Samples from the western region are indicated in red and samples from the eastern region are indicated in blue. Samples without *A. terreus* are dashed to the upper right and samples with *A. terreus* are dashed to the upper left.
