# Supplementary material for: Exploring Factors Driving the Uneven Distribution of Aspergillus terreus in an Austrian Hotspot Region
Source: Microorganisms. 2025 May 27;13(6):1218. doi: 10.3390/microorganisms13061218 (PMC12195318; doi:10.3390/microorganisms13061218)
Supplement: Supplementary file 1 [file microorganisms-13-01218-s001.zip › Figure S2.pdf]

microorganisms

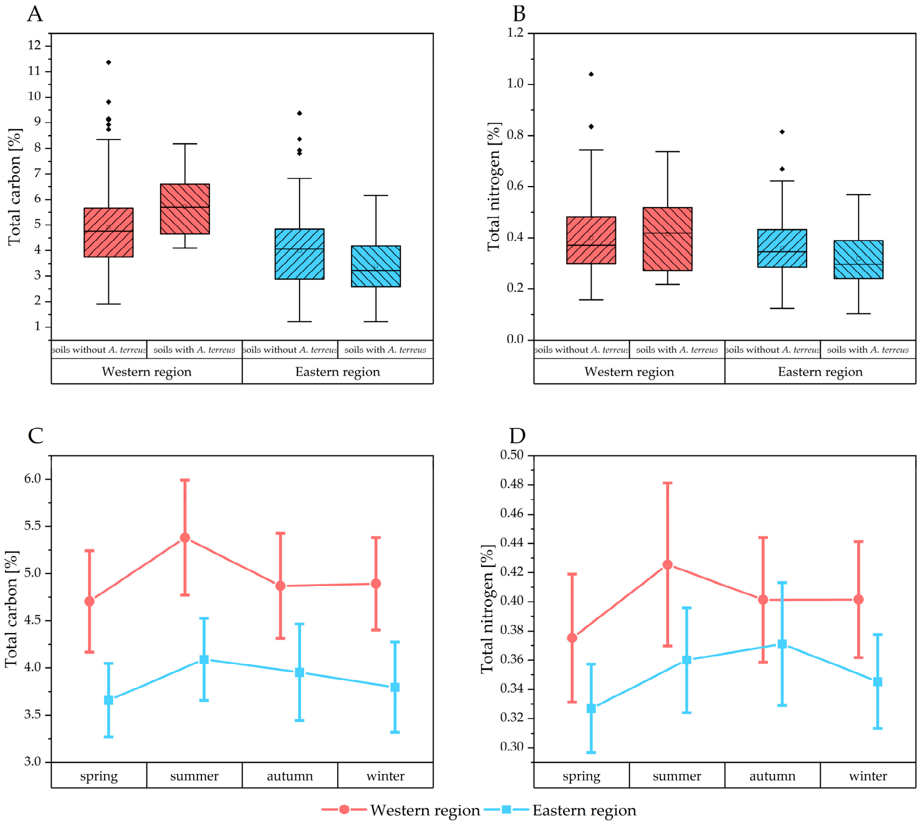

Figure S2. Physicochemical properties of the soil samples including total carbon (C) (A & C) and nitrogen (N) (B & D). Boxes represent 25-75% of values, black lines medians, whiskers 1.5 interquartile ranges, hollow squares means and diamonds outliers (A & B). Data points represent means and error bars represent 95% confidence intervals (C-F). Samples from the western region are indicated in red and samples from the eastern region are indicated in blue.
