# Supplementary material for: Exploring Factors Driving the Uneven Distribution of Aspergillus terreus in an Austrian Hotspot Region
Source: Microorganisms. 2025 May 27;13(6):1218. doi: 10.3390/microorganisms13061218 (PMC12195318; doi:10.3390/microorganisms13061218)
Supplement: Supplementary file 1 [file microorganisms-13-01218-s001.zip › Figure S3.pdf]

microorganisms

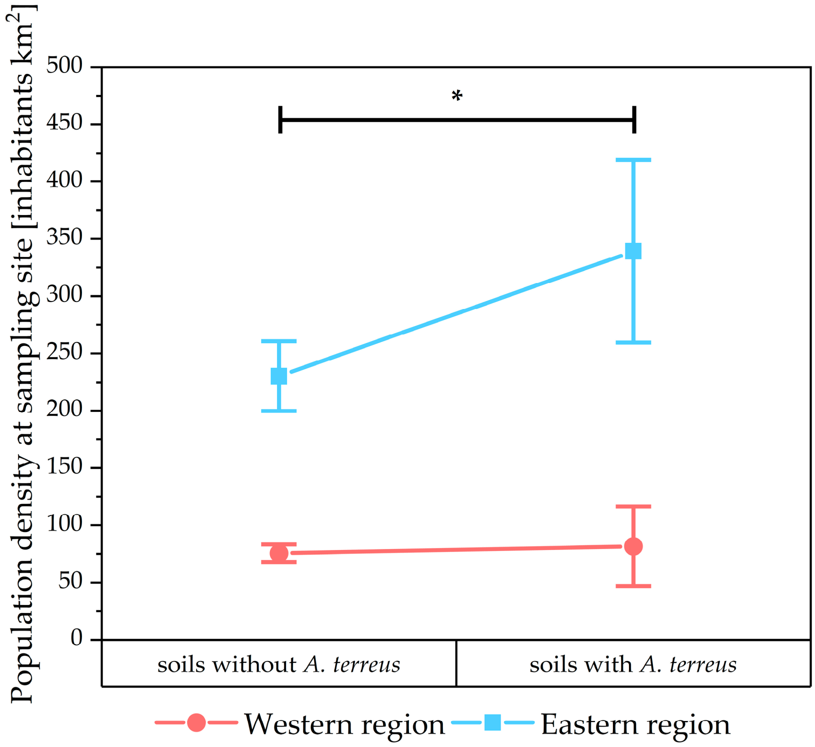

Figure S3. Population density (from STATAtlas [23]) of the sampling sites. Comparison between soils with and without *A. terreus* sorted by sampling region (Mann-Whitney test: $z = -11.38$, $p < 0.05$). Data points represent means and error bars represent 95% confidence intervals. Samples from the western region are indicated in red and samples from the eastern region are indicated in blue.
